# The Health Effects of Heated Tobacco Product Use—A Narrative Review

**DOI:** 10.3390/healthcare13162042

**Published:** 2025-08-18

**Authors:** Małgorzata Znyk, Dorota Kaleta

**Affiliations:** Department of Hygiene and Epidemiology, Medical University of Lodz, Zeligowskiego 7/9, 90-752 Lodz, Poland; dorota.kaleta@umed.lodz.pl

**Keywords:** cells, Glo, health effect, heated tobacco product, human, IQOS, Ploom

## Abstract

One of the most popular currently available tobacco products is the heated tobacco product (HTP), which heats nicotine and other chemical substances into a vapor for inhalation. The aim of the present review was to clarify the effects of exposure to HTP, which currently remain unclear. A literature search of Web of Science, Scopus, ClinicalKey, and PubMed was conducted. The search identified 55 studies on humans and human cells in vitro (mostly independent, i.e., not funded by the tobacco sector) published from February 2021 to May 2025. Studies evaluating the effects of HTP use on the cardiovascular system indicate an increase in blood pressure, heart rate, platelet clot formation, and an enhanced inflammatory response, which is often followed by endothelial dysfunction. Increases in white blood cell counts, pro-inflammatory cytokines, leukocytes, eosinophils, platelets, IL-6, IL-2, IL-8, total NNAL, and 2,3-d-TXB2 were also observed. The studies suggest a positive correlation between HTP use and the occurrence of respiratory diseases, with particular negative effects observed on lung physiology, human bronchial epithelial cells, acute eosinophilic pneumonia, allergies, and asthma. Our findings indicate that the use of HTP is associated with possible adverse effects on the reproductive system. The review also identified new studies on the health effects of HTP use during pregnancy on the fetus, newborn, and mothers. Further research is needed to determine the short-term and long-term health effects of using HTP products.

## 1. Introduction

Recent years have seen the tobacco market undergo dramatic changes [1]. In addition to traditional cigarettes and smoking tobacco, novel tobacco products have enjoyed increasing popularity. A novel tobacco product is defined as “a tobacco product other than a cigarette, pipe tobacco, waterpipe tobacco, roll-your-own tobacco, cigarillo, cheroot, nasal tobacco, chewing tobacco, or oral tobacco” [2]. Innovative tobacco products were approved for sale by the FDA (Food and Drug Administration) in 2019, with the caveat that their approval did not indicate that the products were in any way healthier [3]. In European Union countries, such products have been approved for sale since 2016 [4]. The market for novel tobacco products in the European Union (EU) countries has been regulated by law (Directive 2014/40/EU of the European Parliament and of the Council of 3 April 2014), and good regulatory practices have also been indicated by the WHO (World Health Organization) in the WHO FCTC (Framework Convention on Tobacco Control) document [4].

One of the most popular innovative tobacco products is the heated tobacco product (HTP); these devices produce a vapor containing nicotine and other chemical substances by heating a liquid; the vapor is then inhaled through the mouth. Depending on their characteristics, HTPs can either be classed as a smoking tobacco product or a smokeless tobacco product [5,6].

Heated tobacco products constitute a separate category of nicotine products from electronic cigarettes [7,8]. While they first appeared in the 1960s, they have only received widespread marketing and distribution in recent years [6]. The most popular HTP brands on the market are currently IQOS (I-Quit-Ordinary-Smoking), lil, Ploom, Mok, Glo, and iFuse, currently available in over 60 countries [9,10], which are slowly replacing e-cigarettes [11]. The device includes a tobacco heater insert that contains the nicotine equivalent of approximately 80% of one cigarette, making it harmful and addictive [12,13,14]. The liquid used in HTP is available in a variety of flavors, e.g., menthol, mint, and fruit, which are attractive to both non-smokers and smokers who want to change their current tobacco-related experiences [12].

Due to a combination of misinformation, a lack of understanding about heated tobacco, and gaps in legislation, regulations regarding the marketing of tobacco products have sometimes been poorly enforced [12].

Research in the European Union indicates at least a 10% increase in the volume of HTP sales and more than 2.5% of total sales of tobacco products at the retail level [4]. About 6.5% of EU citizens have used HTPs at least once, 0.7% were daily users, and 1.3% were current users, and HTPs are used more often by former and current smokers and younger people [15]. In fact, young adults aged 18–24 have been found to be the most active users [11]; this has been attributed to intensive marketing in this age group by tobacco companies [16,17].

Such popularity among younger adults may be influenced partly by a loophole in the regulations that does not prohibit their use indoors, as is the case with combustible cigarettes, and partly by the nature of the devices themselves, i.e., their technological attractiveness, novelty, appealing appearance, or low price [18]. The group are also specifically targeted by HTP advertising campaigns [19], which suggest that HTPs are a low-risk product and not a harmful substance [20]. Both young users of HTCs, i.e., vapers, and smokers are more likely to be exposed to advertising than non-smokers, either on websites or social media or in shops selling cigarettes [21,22].

The phenomenon of double smoking is becoming more and more common, i.e., using a traditional cigarette and an HTP or e-cigarette during the day or using an HTP and an e-cigarette at the same time [23,24]. Furthermore, similarly to traditional smoking, the use of HTPs may also entail passive smoking, i.e., inhalation of the aerosol by people near the user [25].

However, such alternative nicotine delivery systems do not appear to represent a healthier alternative to traditional cigarettes and may also encourage traditional smoking [12]. There is currently insufficient evidence that using HTPs is less harmful than smoking traditional tobacco products, nor is there any evidence that smokers who switch from traditional cigarettes to HTPs enjoy any health benefits such as improved overall respiratory function or reduced inflammation. Due to the variety of these products on the market and their short availability, little research has been performed on their long-term adverse health effects, including the risk of developing cancer [12].

To address this current gap in research, the aim of the present article was to determine the effects of exposure to HTPs on human health.

Many studies aimed at assessing the potential impact of tobacco use on health [26] are focused on short-term health effects such as oxidative stress, inflammation, glucose and lipid metabolism, and lung function in smokers and non-smokers [27], with the aim of indicating whether new tobacco and nicotine products pose fewer health risks than smoking [28]. These analyses often employ biomarkers of potential harm (BoPH) and serve as early indicators of the physiological changes caused by tobacco use and may indicate disturbances in biological processes that lead to smoking-related diseases such as chronic respiratory disease and chronic heart disease [29,30]. Commonly used BoPH include interleukin-6 (IL-6), fibrinogen, high-sensitivity C-reactive protein (hs-CRP), F2-isoprostane, white blood cell count (WBC), and forced expiratory volume in one second (FEV1) [31,32]. These indicators are typically higher among smokers compared to those who have never smoked [32]. BoPHs are also used to assess the transition to potentially lower-risk tobacco products or to assess the beneficial effects of quitting smoking [31] and can indicate how quickly physiological functions in the human body can improve after quitting smoking [31].

This review, a continuation of our previous systematic review, includes clinical cases, human studies, and in vitro human cell studies examining the health effects of heated tobacco products. All papers were published between 2021 and 2025.

## 2. Materials and Methods

The article is a narrative review.

### 2.1. Search Strategy

A literature search was conducted by Web of Science, Scopus, ClinicalKey, and PubMed using the following keyword combinations: heated tobacco product, IQOS, Ploom, Glo, health effects, human, and cells. The analysis included peer-reviewed, full-text publications in English published from February 2021 to May 2025. Publications that were published after our previous systematic review were included [33]. Previous publications (published up to January 2021) were excluded from the study because they were included in an earlier review. The literature search includes publications published until the end of May 2025, when the review was completed.

The titles and abstracts were first reviewed, and any original articles, case reports, and systematic reviews consistent with the purpose of the review were subjected to full-text analysis. The search yielded 433 articles, which were then screened for eligibility. The search strategy is presented in Figure S1 (Supplementary Material). Further papers were identified by searching the reference lists of the selected review articles.

### 2.2. Study Selection Criteria

The 433 publications identified in the search were reviewed simultaneously by two reviewers. In the case of disagreement between the two, consensus regarding the included studies was reached through discussion. The articles included by the two investigators were independently reviewed and approved by the third author.

Inclusion criteria: articles describing the association between heated tobacco products and adverse health outcomes in adults, or exposure to heated tobacco products; full-text publications, English-language publications, peer-reviewed original studies, or case reports. To be included in this review, a study had to report on a study population using HTP or at least one individual using HTP (case report); HTP users aged ≥15 years. The corpus also included articles comparing HTP users with e-cigarette users, traditional cigarette smokers, and non-smokers in terms of health effects of exposure.

Exclusion criteria: incomplete text publications, duplicate publications, studies published before February 2021, systematic reviews and narrative reviews, and letters to the editor. Studies that did not report on the association between heated tobacco products and health outcomes, those that exclusively reported HTP use, and those that included a non-HTP-using population aged <15 years were also excluded.

After exclusion, 55 articles describing the adverse health effects of exposure to heated tobacco products were identified and included in the study: these comprised 52 original articles and three clinical case reports assessing the impact of HTPs on health outcomes in humans. The majority of articles identified in the first search (90%) did not meet the inclusion criteria because they did not address the health effects of HTPs in humans. The studies included in the narrative review are given in Table S1 (Supplementary Material).

The corpus included both in vivo human studies and in vitro studies on human cells; one of the in vitro studies was industry sponsored.

### 2.3. Data Extraction and Quality Assessment

The extracted data included the following elements: author, year, study funding, country, study design, sample size, study objective, results, and quality assessment. The information was extracted from each study by the first author, who prepared a narrative summary. The summary was then reviewed independently by the second author. Discrepancies were resolved by referring to the original articles and case reports.

The extracted data were subjected to quality assessment by two investigators (MZ, DK). Non-randomized studies were assessed using the Newcastle-Ottawa Scale (NOS) and a modified scale adapted for cross-sectional studies [34]. Randomized controlled trials (RCTs) were evaluated using the Cochrane Risk of Bias for Interventional Studies [35]. Data were summarized descriptively using a narrative synthesis of the findings.

## 3. Results

### 3.1. The Impact of Heated Tobacco Products on Human Health in In Vivo Human Studies

#### 3.1.1. Respiratory System

The association between exposure to heated tobacco products and respiratory disease has been investigated in independent human studies and clinical case reports [36,37,38,39,40,41,42,43,44,45]. Three of the human studies were conducted in Japan [39,41,42], and one each in Poland, Kazakhstan, Italy, and Germany [36,37,38,45]. The review included three clinical cases of HTP users: one from Qatar [40], one from Kaunas [43], and one from Turkey [44].

*Advantages* *of using HTP*

NTV-only (novel tobacco vapor) users were characterized by more favorable BoPH than traditional cigarette smokers [39]. Levels of 4-(methylnitrosamino)-1-(3-pyridyl)-1-butanone (NNK), a carcinogen, were significantly more favorable in NTV-only users compared to CC smokers. Plasma cotinine levels were significantly lower in the NTV group compared to the CC group (−73.0%, *p* < 0.0001), as were NNAL levels (−94.3%, *p* < 0.0001). Significantly higher NNAL levels were observed in the NTV group compared to the NS group (207%, *p* < 0.0001) [39].

A study including a four-year follow-up found HTP users to suffer significantly less decline in lung function than traditional cigarette users (*p* < 0.0001) and to perform better in other functional outcomes, *viz.* CAT (COPD Assessment Test) (*p* < 0.0001) and physical characteristics [38,46]. Elsewhere, COPD patients who had significantly reduced cigarette smoking or abstained altogether by switching to HTP displayed a significant reduction in annual COPD exacerbations and displayed improved 6MWD (6 min walking distance) (*p*  < 0.01) and CAT scores at all three studied time points (*p*  < 0.01); they also achieved improvements in respiratory symptoms, quality of life, exercise tolerance, and exacerbation frequency [45]. The reduction in annual COPD exacerbations was from 2.1 (±0.9) at baseline to 1.4 (±0.8), 1.2 (±0.8), and 1.3 (±0.8) at 12-, 24-, and 36-month follow-up (*p* < 0.05) [45].

*Disadvantages* *of using HTP*

Three clinical cases of users of HTPs who switched from traditional cigarettes indicate lung damage induced by HTP.

In one case, a 40-year-old man who switched from traditional cigarettes to IQOS showed incidental abnormalities: ground-glass opacities and pulmonary nodules on computed tomography, mild peripheral eosinophilia on bronchoscopy, and abnormalities on chest radiography. The patient reported no systemic complaints. Discontinuation of HTP use resulted in radiographic improvement and resolution of symptoms [40].

Another case concerned a 22-year-old woman who presented with cough, dyspnea, and fever and was diagnosed with acute eosinophilic pneumonia (AEP). The interview revealed a two-week history of HNBC use (15 cigarettes per day before the onset of symptoms). The patient presented bilateral patches of pulmonary infiltrate on radiographs, eosinophilia, and multifocal bilateral patchy consolidations with multiple small nodular ground-glass opacities and interlobar septal thickening on computed tomography. Intravenous followed by oral methylprednisolone for 14 days resulted in the resolution of symptoms. A follow-up visit after six months confirmed cessation of smoking and no recurrence of AEP [43].

In the third case, a 56-year-old man who had switched from traditional cigarettes to IQOS was admitted with shortness of breath and sudden chest pain. Lung changes were identified that could be related to HTP use for 2.5 years: fibroatelectatic changes in the lung lobes, pleural atelectasis, pleural effusion in the right upper lobe, as well as extensive anthracosis around the lung, lymphoid aggregation in a nodular form, fibrous material resembling a hyaline membrane in the lung alveoli, interstitial organization, and type 2 pneumocyte hyperplasia. Examination revealed subacute lung injury with exogenous lipid material. The publication does not describe the treatment results [44].

The children of mothers using HTP during pregnancy have been found to be at greater risk of allergies, together with atopic dermatitis, rhinitis, conjunctivitis, and clinical diagnoses of asthma [41]. The prevalence of allergy among offspring of women who smoked HTP during pregnancy was 15.2% (PR = 1.98, 95% CI 1.28–3.05) [41].

Dual users have been found to demonstrate lower FEV1 (forced expiratory volume in 1 s). It was found that previous smokers who had been using HTP for a mean period of 1.7 years after switching from traditional cigarettes exhibited a similar decrease in FEV 1 as cigarette-only smokers [42]. Also, novel tobacco vapor (NTV) users were found to have lower FEV1 and %FEV1 (% predicted FEV1) compared to never-smokers (−4.2%, *p* = 0.0261; −3.3%, *p* = 0.0439, respectively) [39].

However, the consumption of substitute products and cigarettes has also been found to significantly increase resistance and obstruction of small airways [36], and five minutes of exposure to HTP was sufficient to significantly decrease F_ENO_ (fraction of nitric oxide in exhaled air) (from 12.8 ± 5.5 ppb to 11.2 ± 5.3 ppb, *p* < 0.01) [37].

#### 3.1.2. Cardiovascular System

The relationship between exposure to HTP and cardiovascular disease has been investigated in independent studies [36,37,38,39,47,48,49,50,51,52,53,54,55,56,57,58]. Three of these were conducted in Poland [37,48,51], three in Japan [39,49,50], three in Germany [36,52,55], two in Sweden [47,54], one in Italy [57], one in Kazakhstan [38], one in Greece [58], and one in Korea [53].

*Advantages* *of using HTP*

Exclusive NTV (novel tobacco vapor) users demonstrate favorable BoPH (biomarkers of potential harm) levels compared to CC (conventional cigarette) smokers. Significant differences were observed between NTV and CC with regard to TG (22.8% lower in NTV), HDL-C (+13.9%), sICAM-1 level (−12.4%), and WBC counts (−17.8%) [39]. Interestingly, smoking HNBC appears to have less harmful effects on vascular and cardiac functions than traditional cigarettes. Switching to HNBC improved CO (exhaled carbon monoxide), FMD (flow-mediated dilation), CFR (coronary flow reserve), TAC (total arterial compliance), GLS (global longitudinal strain), GWW (wasted myocardial work), MDA (malondialdehyde), and TxB2 (thromboxane B2) compared to traditional cigarettes (differences of 10.42 ppm, 4.3%, 0.98, 1.8 mL/mmHg, 2.35%, 19.72 mmHg%, 0.38 nmol/L, and 45 pg/mL, respectively, *p*  <  0.05) [58].

*Disadvantages* *of using HTP*

The risk of hypertension has been shown to increase with the intensity of HTP use [50]. In the model adjusted for sex, age, and job position, exclusive HTP users had a higher risk of hypertension (HR 1.26, 95% CI 1.13–1.42, *p*  <  0.001), followed by dual users (HR 1.21, 95% CI 1.02–1.43, *p*  =  0.03) and exclusive cigarette smokers (HR 1.20, 95% CI 1.08–1.34, *p*  <  0.001) compared to never smokers [50].

Other studies have confirmed increased blood pressure [36,37,38,54,55], increased arterial stiffness [36,52,54,55], increased heart rate [37,54], increased pulse wave velocity, and platelet clot formation [54] in people using HTPs. In addition, like traditional cigarettes, HTP use has been shown to increase the risk of nicotine-related cardiovascular stress and thus a higher chance of cardiovascular disease [36].

Men using HTPs exhibited significantly higher levels of IL-8 (interleukin 8) compared to controls (6.86 vs. 3.95, *p* = 0.01), with the level of IL-8 being positively correlated with the daily number of heated tobacco pieces (r = 0.37, *p* = 0.01) [51]. Similarly, HTP use was associated with increased IL-8, IL-6, and IL-2 levels (*p* < 0.05), as well as higher white blood cell (WBC), leukocyte (*p* < 0.05), and eosinophil levels (*p* < 0.01), and an increase in the inflammatory response (*p* < 0.01), followed by endothelial dysfunction [52].

The use of HTPs containing nicotine has also been found to potentially pose a significant vascular risk. After short-term inhalation of HTP with nicotine, a significant increase in platelet- and endothelial-derived EVs (extracellular vesicles) was noted, suggesting acute vascular stress (*p* < 0.001). The increase in platelet- and endothelium-derived extracellular vesicles is consistent with responses to acute vascular injury, similar to e-cigarette and traditional cigarette use [47].

A study on HNBCs (Heat-Not-Burn cigarettes) found their use to have deleterious effects on oxidative stress and platelet activation. HNBCs were less deleterious than traditional combustion cigarettes (TCCs) when P-selectin and Nox2-dp (Nox2-derived peptide) were targeted, but they had similarly negative effects on reactive oxygen species (ROS) generation and vascular reactivity. HNBCs and TCCs were similarly deleterious on sCD40L (soluble CD40 ligand), H_2_O_2_ (hydrogen peroxide), NO (nitric oxide), cotinine, platelet aggregation, and FMD (flow-mediated dilation) [57]. HNBCs had similar detrimental effects on platelet activation in men and women (sP-selectin: *p* = 0.33; platelet aggregation *p* = 0.87) and oxidative stress (H_2_O_2_: *p* = 0.49; sNox2-dp: *p* = 0.31) [57].

IQOS users showed significantly higher platelet levels compared to non-smokers and cigarette smokers (290.27 vs. 256.33 vs. 267.14 × 10^3^/μL, respectively, *p* < 0.05) [48].

A positive dose-response relationship has been noted between WBC count and the number of HTPs, or traditional cigarettes, consumed [53].

Significantly lower HDL-C (high-density lipoprotein cholesterol) levels were observed in exclusive HTP compared to non-smokers; however, they exhibited higher levels than exclusive cigarette smokers. Similar HDL-C levels were observed for dual users and exclusive cigarette smokers [56]. Pooled ORs (95% CI) of low HDL-C were 1.25 (1.09–1.43), 2.02 (1.76–2.32), and 2.09 (1.88–2.32) for HTP-only users, dual users, and exclusive cigarette smokers, respectively [56].

HTP users were found to exhibit similar profiles of biomarkers involved in glutamate metabolism as cigarette smokers. These biomarkers are associated with the possible onset of atherosclerosis and endothelial dysfunction. Furthermore, HTP use appears to influence glutamate metabolism, which may contribute to the development of cardiovascular disease. The HTP users were found to be similar to the tobacco smokers, indicated by only minimal differences between them for five metabolites: glutamate (1.10; 95% Cl 0.80–1.53), arginine (1.03; 95% Cl 0.74–1.44), ornithine (0.88; 95% Cl 0.64–1.22), citrulline (0.83; 95% Cl 0.60–1.15), and trigonelline (1.04; 95% Cl 0.79–1.36). (fold change; 95% confidence interval) [49].

Exclusive NTV users also display significantly higher levels of total NNAL (4-(methylnitrosamino)-1-(3-pyridyl)-1-butanol) (207%, *p* < 0.0001), cotinine, and 2,3-d-TXB2 (2,3-dinor thromboxane B2) (+18.1%, *p* = 0.0312) compared to never-smokers. The levels for plasma cotinine were significantly lower in the NS (−99.7%, *p* < 0.0001) and in the NTV (−73.0%, *p* < 0.0001) compared to the CC [39].

#### 3.1.3. Nervous System

*Disadvantages* *of using HTP*

One independent study in humans conducted in the Republic of Korea found HTP users to have the highest percentage of depressed mood and anhedonia. Dual users and HTP users were more likely to experience moderate to severe depressive symptoms [59].

Anhedonia was reported at a high rate in dual users (9.9%) and in HTP-only users (11.5%) (χ^2^ = 7.13, *p* < 0.001). Feelings of low well-being were reported in 7.1% of dual users and 4.9% of HTP-only users (χ^2^ = 68.23, *p* < 0.001). Depressed mood was reported in 3.6% of dual users and 7.5% of HTP-only users, with HTP-only users having the highest rate (χ^2^ = 5.72, *p* = 0.001) [59].

#### 3.1.4. Oral Cavity

The relationship between exposure to heated tobacco products (HTPs) and oral diseases has been investigated in human studies [60,61,62,63,64,65,66,67]. Three of these studies were conducted in Croatia [61,62,65], two in Japan [66,67], two in Poland [63,64], and one in Italy [60]. All of these studies are independent studies (not sponsored by the tobacco industry), apart from one from Japan [67].

*Advantages* *of using HTP*

Gupta et al. found the use of HTPs to potentially be associated with whiter teeth, which is of interest to smokers concerned with dental aesthetics. The difference in tooth whiteness in HTP users was visually noticeable, and exclusive HTP use was associated with better results on tooth color measures compared to smokers. The whiteness index for dentistry (WID) was 13.4 for current smokers and 17.8 for HTP users [60].

Mišković et al. report that exposure to THS (tobacco heating system) aerosols containing nicotine has a less harmful effect on periodontal tissues (lower periodontal PD and CAL indices) than smoking traditional cigarettes. THS users demonstrated poorer CAL and PD than nonsmokers (*p* > 0.05). THS users had lower CAL and PD values than smokers, but these differences were significant only for CAL (r = 0.383, *p* = 0.011) [61].

Interestingly, Pouly et al. report that switching to THS by people with generalized chronic periodontitis can reduce the risk of smoking-related diseases [67].

*Disadvantages* *of using HTP*

An increased incidence of genotoxic and cytotoxic damage was identified in users of non-combustible alternatives, similar to smokers of traditional cigarettes, with a harmful effect on the oral mucosa. These users also demonstrated increased numbers of pyknotic cells (*p* ≤ 0.001) and higher values for all tested parameters in the micronucleus test (*p* < 0.05) (except the number of cells with micronuclei) compared to non-smokers [62].

Zięba et al. found the use of heat-not-burn (HnB) products, e-cigarettes, and smoking traditional cigarettes to be responsible for changes in the local immune response in saliva [63]; in addition, HnB products and e-cigarettes appear to have a similar mechanism of action on the immune system of unstimulated saliva [63].

Compared to smokers of traditional cigarettes, HnB users had significantly lower levels of certain salivary cytokines IL-16 (↓61%, *p* = 0.0463) and HGF cytokines (↓54%, *p* = 0.0004), and the chemokines Gro-α (↓65%, *p* = 0.0002), MCP-1 (↓47%, *p* = 0.0253), SCF (↓63%, *p* = 0.0286), MIG (↓48%, *p* = 0.0081), and IP-10 (↓52%, *p* = 0.0027). They also presented lower levels of growth factors G-CSF (↓57%, *p* = 0.0019) TRAIL (↓55%, *p* = 0.0005) [63].

Other studies indicate that smoking was found to increase the MDA (malondialdehyde) and 4-HNE (4-hydroxynonenal) content in saliva (*p* < 0.0001) and decrease its lipid concentration (*p* < 0.0001) [64]. The HTP users also demonstrated significantly lower stimulated salivary 4-HNE levels compared with traditional cigarette smokers (*p* = 0.0002) [64].

Sever et al. suggest that THS has a similar effect on halitosis and SFR (salivary flow rate) as smoking conventional cigarettes: both were associated with significantly lower SFR and a significantly higher prevalence of intraoral measures, halitosis, and dry mouth [65]. Significant differences between smoking groups were found for SFR (*p* < 0.001; ε^2^ = 0.0331), intraoral symptoms (*p* < 0.001; ε^2^ = 0.507), halitosis (*p* < 0.001; ε^2^ = 0.624), and dry mouth (*p* = 0.021; ε^2^ = 0.363) [65].

Mori et al. suggest that HnB use may be associated with significantly lower salivary secretion (*p* < 0.01) and lower Lys (lysozyme secretion indicators) and Lac (lactoferrin secretion indicators) levels (all *p* < 0.01) in oral saliva compared to non-smokers [66].

#### 3.1.5. Reproductive System

Three independent studies examined the impact of HTP use on the human reproductive system [68,69,70]. Two of these studies were conducted in Italy [68,69] and one in Japan [70].

*Disadvantages* *of using HTP*

Incognito et al. found pregnant mothers who smoked HTP were more at risk of preterm birth, similarly to cigarette smokers (CS) (17% and 20%, respectively) [68].

Galanti et al. report that the use of alternative cigarette devices negatively affected fertility, leading to reduced ovarian quality and reduced ovarian reserve; it also negatively affected the quality and quantity of oocytes collected in ICSI (intracytoplasmic sperm injection) cycles. Among women, the number of collected oocytes was lower in the smoker group compared to the non-smoker group (5.21 ± 0.9 vs. 6.55 ± 3.5, *p* < 0.001). HnB users also demonstrated reduced anti-Mullerian hormone (AMH) levels (*p* < 0.05) [69].

Hosokawa et al. indicate that HTP smoking may be associated with an increased risk of SGA (small for gestational age) in pregnant women: those who smoked HTP exclusively during pregnancy were at higher risk of SGA than those who had never smoked [70]. The odds of SGA were significantly increased in HTP smokers (OR = 2.70; 95% CI: 1.14–6.40). The prevalence of SGA was 5.9% among exclusive HTP smokers and 2.7% among never smokers; the prevalence of preterm birth was 8.8% and 5.5% among the respective groups [70].

#### 3.1.6. Metabolic System

Three independent studies examined the effect of HTP use on the metabolic system in humans: one from South Korea [71], one from Japan [72], and one from Kazakhstan [46].

*Disadvantages* *of using HTP*

A study by Jee et al. found HTP users to have an increased risk of metabolic syndrome compared to never smokers. The risk of metabolic syndrome was 68% higher (HR = 1.68; 95% CI: 1.25–2.26) in current HTP users compared to those who had never smoked. This risk was also higher than that observed in users of traditional cigarettes. This increase was particularly apparent among those using HTP for more than three years. In addition, the risk of metabolic syndrome was 2.17 times higher among current HTP users and former users who had taken them for more than three years, who did not smoke traditional cigarettes, than among non-users of HTP (HR = 2.17; 95% CI: 1.31–3.62) [71].

Hu et al. report that exclusive HTP users demonstrated higher odds of developing diabetes and prediabetes than never smokers (pooled odds ratio 1.68; 95% CI: 1.45–1.94 and 1.36; 95% CI: 1.25–1.47, respectively). Dual users had similar odds (pooled odds ratio 1.93; 95% CI 1.63–2.29 and 1.26; 95% CI 1.13–1.39, respectively). The exclusive and dual HTP users also exhibited significantly higher fasting glucose and HbA1c levels compared to never smokers [72]. Similarly, HTP users demonstrated poorer fasting blood glucose levels during a four-year follow-up. The estimated marginal mean HTP users by 48-month follow-up of glucose was 5.717 (95% CI: 5.554–5.879), and at the beginning of the follow-up, 5.359 (95% CI: 5.206–5.512) [46].

#### 3.1.7. Biomarkers of Exposure and Biomarkers of Effect

Three studies described biomarkers of exposure [73,74,75] and biomarkers of effect; two of them were conducted in the UK [74,75] and one in China [73].

*Advantages* *of using HTP*

Chu et al. report lower BoE (biomarkers of exposure) levels in the HTP device group compared to the control group (smoking traditional cigarettes); however, no significant changes in BoBE (biomarker of biological effect) levels were observed in either group. HTP users experienced reduced cravings and withdrawal symptoms [73]. BoE levels decreased by 26.4% to 71.4% in the HTP device group. Mean scores for satisfaction and harm reduction beliefs were 7.4 and 8.7, respectively (on a scale of 1–10) [73].

A one-year study found that individuals who quit smoking completely or switched to HTP demonstrated several favorable changes in BoPH levels. After 360 days, participants who switched from smoking to HTP use and participants who quit smoking had significant and sustained reductions in BoE levels. The participants who continued smoking demonstrated unfavorable changes in the levels of BoPHs, such as soluble intercellular adhesion molecule-1 [74]. BoE reductions for NNK and NNN (*N*-nitrosonornicotine) from baseline to 180 days ranged from 38% to 43% in the HTP group and from 73% to 75% in the quit group. The NNK reductions were greater than those reported in other short-term studies, including an HTP group and a quit group, with similar reductions for NNN [74].

In another study, most BoE levels in HTP users were observed to have decreased significantly over 180 days, becoming similar to those in nonsmokers. Significant changes were noted in BoPH, i.e., total 4-(methylnitrosamino)-1-(3-pyridyl)-1-butanol, 8-epi-prostaglandin F2α type III, fractional exhaled nitric oxide concentration, and white blood cell count, which were consistent with better health [75].

#### 3.1.8. Molecular Genetic Effects

*Disadvantages* *of using HTP*

One molecular study conducted in Japan describes the genetic effects of HTP use in users who had stopped smoking conventional cigarettes for less than two years. All demonstrated abnormal DNAm (DNA methylation) and transcriptome profiles, but to a lesser extent than in conventional cigarette smokers. *LRRN3* and *GPR15* (DNAm biomarker genes) were more hypomethylated in HTP users, while *GPR15* expression was significantly increased in all groups (combustible tobacco smokers, HTP users, and past smokers) compared to never smokers [76].

#### 3.1.9. Secondhand Exposure

*Disadvantages* *of using HTP*

One study conducted in Japan found that passive exposure to HTP aerosol was associated with asthma-like respiratory symptoms, asthma attacks (PR 1.49, 95% CI: 1.21–1.85), and persistent cough (PR 1.44, 95% CI: 1.21–1.72) [77].

The health effects of heated tobacco product use in human studies are presented in Table 1.

### 3.2. The Impact of Heated Tobacco Product Use on Human Health in In Vitro Studies on Human Cells

The review included 13 studies describing the health effects of exposure to aerosols of heated tobacco products in human cells in vitro [78,79,80,81,82,83,84,85,86,87,88,89,90]. Four were conducted in Japan [80,83,87,89], four in Italy [78,79,86,90], two in France [81,82], two in Germany [85,88], and one in Sweden [84]. Almost all studies were independent studies [78,79,80,81,82,83,84,85,86,88,89,90], except one [87].

*Advantages* *of using HTP*

Exposure to HTP aerosols was found to reduce the viability of fibroblasts, which highlights clear advantages without showing mutagenic potential or cytotoxicity compared to conventional cigarettes. However, the aerosols exerted bactericidal effects against Klebsiella pneumoniae and Streptococcus pneumoniae at higher concentrations and bacteriostatic effects at lower concentrations. HTP aerosols have demonstrated antibacterial properties, inhibiting the growth of *K. pneumoniae* and *S. pneumoniae*. While initially inhibiting bacterial growth may seem beneficial in reducing pathogen burden, this may disturb the balance of the microbial community, causing the bacteria to transition from a commensal to a pathogenic state, increasing the risk of respiratory infections [78].

The vapor from HTPs may have a different toxicological profile against microglial cells compared to cigarettes. Compared to traditional cigarettes, HTP showed reduced ROS production, lower microglial toxicity, reduced lipid peroxidation, and mitochondrial dysfunction [79].

Other studies found HTP to have a less harmful effect on hPDL (human periodontal ligament cells) than CS (cigarette smoke). The findings suggest that HTPs, or electronic cigarette vapor, may offer a reduced risk to periodontal health than conventional cigarettes. HTP-treated cells showed significantly reduced cell numbers at 48 and 72 h (*p* < 0.001) compared to control cells and showed a noticeable decrease in cell metabolic activity at 48 h (*p* < 0.001) [88].

Exposure to IQOS extracts increased the viability and migration of both keratinocytes and gingival fibroblasts, as well as the numbers of cells in the S and G2/M phases (*p* < 0.001 for both and *p* < 0.05 for both, respectively); it also increased p53 expression and decreased Bcl2 and p21 expression in the fibroblasts and increased Bcl2 expression and decreased p53 expression in the keratinocytes. Although the IQOS extract influenced cell cycle and proliferation, it does not appear to be toxic to oral keratinocytes and fibroblasts, as it did not change either cell morphology or survival rate [90]. p53 expression increased twofold in human gingival fibroblasts in the undiluted extract and 3.6-fold in the 6.25% diluted HTP extract (*p* < 0.001), compared with the control group. p21 levels decreased approximately twofold in both cases (*p* < 0.001), and Bcl2 expression levels decreased approximately twofold in the undiluted extract and fivefold in the diluted extract (*p* < 0.001). In keratinocytes, p53 expression decreased (approximately two- and five-fold higher, respectively), and Bcl2 expression increased (approximately four- and three-fold higher, respectively) [90].

*Disadvantages* *of using HTP*

Both HTPs and traditional tobacco demonstrated cytotoxic effects against OSCC (oral squamous cell carcinoma) cells. Exposure to CSE (cigarette smoke extract) from both sources induced cell apoptosis, increased ROS levels, induced p38 phosphorylation, and led to an increase in intracellular Ca^2+^ (calcium ions) concentration [80].

Similarly, both HTP and cigarette 3R4F emissions affected the profiles of exogenous compounds in BEAS-2B (human bronchial epithelial cells) cells, including one carcinogen; they also influenced the profiles of endogenous metabolites from, inter alia, lipid metabolism, energy metabolism, and oxidative stress pathways. These effects were observed at higher doses for HTP (60 and 120 puffs) than for cigarettes (two and four puffs). The results suggest that cigarettes and HTP have higher acute toxicity compared to e-cigarettes [81].

Another study found HTP to be less cytotoxic than conventional cigarettes in BEAS-2B cells. Even so, HTP and cigarettes both induced oxidative DNA damage and significantly increased chromosomal aberrations and DNA strand breaks [82].

Heated tobacco products were found to have similar cytotoxic effects as conventional cigarettes, with both being genotoxic and causing double-strand breaks and toxicogenomic damage; both also were found to inhibit ATR-CHK1 and MDC1 repair pathways in the oral mucosa [83]. In addition, a study of the pulmonary toxicity of HTP smoke found it to cause DNA damage, oxidative stress, ferroptosis, and lipid peroxidation [84]. The secretion of pro-inflammatory cytokines IL4 (*p* = 0.01) and IFNγ (*p* < 0.0001) increased, and the secretion of IL13 decreased (*p* = 0.02) after exposure to HTP smoke [84].

A comparison of the effect of HTP, e-cigarettes, conventional cigarettes, and pure nicotine exposure on endothelial function found HTP treatment to induce NQO1 and HMOX1 at nicotine doses ≥1.68 μg/mL. HTP also reduced VCAM1 (vascular cell adhesion molecule 1) expression in a dose-dependent manner and activated antioxidant or pro-inflammatory patterns [85].

Some data suggests that chronic HTP users may be at elevated risk of cardiac fibrosis. Atrial fibrosis plays a key role in the development of atrial fibrillation in chronic smokers of traditional cigarettes. Human cardiac stromal cells exposed to the serum of chronic exclusive HTP smokers showed increased mTOR pathway activation and demonstrated lower beneficial paracrine effects on cardiomyocytes and endothelial cells [86].

Other findings indicate that HTP and traditional cigarettes may alter gene transcription and CpG methylation in lung epithelial cells, with the HTP extract affecting gene expression to a lesser extent than a reference cigarette extract. It also significantly affected mRNA expression and methylation of the CYP1A1 (cytochrome P450 family 1 subfamily A member 1) promoter, which is associated with carcinogenic risk [87].

However, long-term HTP stimulation was also found to influence the differentiation and keratinization of gingival epithelial cells, with the results suggesting that habitual use may be a risk factor for oral mucosa diseases. Gene ontology (GO) analysis showed that genes related to cornification and keratinization were induced by long-term HTP stimulation [89].

The included in vitro studies of the health effects of HTP use are summarized in Table 2.

## 4. Discussion

The debate about the health effects of using heated tobacco products (HTPs) continues [91]. However, our present findings indicate that these products are not safe for health, with a considerable body of evidence indicating that HTP use has adverse cardiovascular and respiratory effects. More specifically, their use leads to an acute increase in arterial stiffness and nicotine-related cardiovascular stress similar to conventional cigarettes, and thus increased cardiovascular risk [36].

Studies also indicate HTP use to be associated with an increase in blood pressure, heart rate, platelet clot formation, and an enhanced inflammatory response, which is often followed by endothelial dysfunction. Increases have also been observed in white blood cell counts and the levels of proinflammatory cytokines, leukocytes, eosinophils, platelets, IL-6, IL-2, IL-8, total NNAL, and 2,3-d-TXB2. Our present findings suggest that HTP use may also correlate positively with the occurrence of respiratory diseases, i.e., with negative effects on lung physiology, human bronchial epithelial cells, acute eosinophilic pneumonia, allergies, and asthma. In addition, HTP users exhibit lower levels of FEV1 and %FEV1 and BoE compared to never-smokers. However, several BoPHs have also been found to return to favorable levels in smokers who switch to HTPs or quit smoking.

In addition, the use of HTPs appears to be associated with possible adverse effects on the reproductive system, and HTP use during pregnancy appears to have potential adverse effects on the fetus, newborn, and mothers. Further research in this area is warranted.

Several in vitro studies on human bronchial epithelial cells and coronary artery endothelial cells indicate that HTP aerosol is less toxic than smoke from traditional cigarettes, with fewer genotoxic, mutagenic, and cytotoxic effects. Nevertheless, HTPs may exert prothrombotic and proatherogenic effects by increasing oxidative stress, promoting platelet activation, and causing endothelial dysfunction [92].

The introduction of HTPs and other innovative tobacco products to the market has resulted in a shift from single-use tobacco products to multi-use products. Heated tobacco products may be an alternative for heavy smokers who are trying to quit smoking and for whom comprehensive treatment of nicotine addiction and smoking has failed [93]. Indeed, the use of traditional cigarettes or e-cigarettes is the most important factor determining the use of HTP worldwide [94,95,96,97,98].

An HTP aerosol allows much faster nicotine absorption than patches, nicotine gum, or lozenges, as the drug is delivered to the throat and lungs rather than the skin or buccal mucosa [99]. The speed at which nicotine is absorbed may be one of the key factors determining addiction [100]. As such, HTPs may be a better substitute for smoking than nicotine replacement therapy [101].

Furthermore, as HTPs do not produce odor, ash, or visible smoke, they may have greater social acceptability than traditional cigarettes [102].

HTP use may negatively impact public health by encouraging non-smokers to smoke or by encouraging them to start smoking or relapse [103].

Available research does not clearly indicate whether HTP can help smokers of traditional cigarettes quit smoking [101,104].

In a study conducted in Japan, current smokers of traditional cigarettes who intended to quit were more likely to use IQOS than those who did not intend to quit [97]. Also, it appears that the simultaneous use of HTPs and e-cigarettes may be associated with an increased number of attempts to quit traditional cigarettes [93].

One study found dual and traditional cigarette-only smokers to demonstrate similar quitting behavior. In contrast, HTP-only users had fewer previous quit attempts and were less likely to be ready to quit. This may be due to the convenience of HTPs and the belief that HTPs are less harmful than traditional cigarettes [105].

Dual users, i.e., conventional cigarettes and e-cigarettes, and triple users, i.e., traditional cigarettes, HTPs, and e-cigarettes, were more likely to attempt to quit smoking conventional cigarettes compared to traditional cigarette-only users [106,107]. On the other hand, dual users of traditional cigarettes and e-cigarettes were more likely to use HTP more frequently and intensely than traditional cigarette users who did not use e-cigarettes. Daily e-cigarette users had the highest risk of frequent and heavy HTP use. Triple use was associated with a lower likelihood of quit attempts compared to combustible cigarette-only users. HTP use was less likely to replace conventional cigarette use and promoted quit attempts [108].

However, a study of Korean teenagers found that using HTP was unlikely to make them quit smoking [109]. In addition, it has been shown that people who use HTP, e-cigarettes, and traditional cigarettes are less likely to quit smoking traditional cigarettes [93]. Daily HTP use appears to be associated with an increased rate of quitting smoking conventional cigarettes, while discontinuation of HTP use is significantly associated with relapse to conventional cigarette smoking among former HTP users within one to five years [110]. Former smokers report that HTP use helped them quit smoking, which is also consistent with studies described earlier that suggest HTP may be useful in facilitating a complete transition from smoking cigarettes [111].

Other studies have found HTP use to be associated with the initiation or relapse of smoking [112].

Although sales of conventional tobacco products are steadily declining, alternative tobacco products are becoming increasingly popular, especially among teenagers [113]. A significant risk is posed by the use of HTPs among adolescents and young adults, as this may be a gateway to future use and addiction to other tobacco products [93]. As such, the growing popularity of HTPs among young people may pose a threat to their health [93,114].

It is important to remember that nicotine is highly addictive, and exposure to nicotine is harmful to developing brains. It is also harmful to adults, pregnant women, and developing fetuses [9,115].

Like traditional cigarettes, HTP use can cause psychological effects, such as nicotine addiction, and influence behaviors related to tobacco use and attitudes toward smoking. For many users, HTP can provide a sense of stress relief and calm, similar to traditional cigarettes. Additionally, like smoking, it can cause symptoms of depression [12,59].

Therefore, there is a need to better educate potential users about the potential harm associated with HTP use. While such initiatives should be directed towards younger people, they should not just be restricted to this age group. Furthermore, there is a need to update tobacco control policies to accommodate the risk posed by newer products [93]. For example, the widespread sale and marketing of HTPs could be restricted [116], or taxes could be placed on the sale of these products to make them less affordable for teenagers [113]. There is also a pressing need to more tightly control the advertising, sponsorship, and promotion of HTPs.

Indeed, while evidence suggests that HTP use is likely to be significantly less harmful than smoking [33,99,101,117,118,119,120,121], their use nevertheless has negative health effects.

Our review also suggests that smokers who switch to HTP may receive beneficial effects by reducing the risk of smoking-related diseases. Indeed, smokers who switch to HTP or who completely quit tobacco or nicotine use demonstrate similar BoE profiles. However, further research is needed to more precisely define this risk reduction potential in smokers who switch to HTP, including assessment of disease endpoints such as cardiac or respiratory events.

A strength of the study is that it brings together the latest research on the effect of HTPs on human health. It includes a range of different studies with different sample sizes and methodologies, which may contribute to the variability of the results. However, much of the data were self-reported, which may be subject to bias: participants may have provided inaccurate information about their tobacco use habits. Also, most of the studies are cross-sectional and observational, making it difficult to establish causal changes at the individual or population level.

Many of the studies included in the present review, published between 2021 and 2025, are independent studies, and their number has increased in recent years. Nevertheless, the body of evidence regarding the health effects of long-term HTP use remains insufficient [115]. As such, long-term studies are needed to provide evidence regarding the long-term potential health effects and safety of these products; these can take the form of case-control studies with HTP users and controls or prospective cohort studies. Such research should include larger study samples and standardized research methods.

The available literature is also limited by the heterogeneity of research designs, reliance on in vitro models, and potential conflicts of interest related to research funding by the tobacco sector. There are also no long-term studies that can unequivocally rule out or confirm a link between HTP use and cancer development.

Although many molecular and cellular mechanisms have been described for the harmful effects of tobacco smoking on the cardiovascular and respiratory systems, a significant knowledge gap exists regarding the mechanisms and extent of damage associated with chronic HTP use. Therefore, to provide a deeper scientific insight into the observed associations, further research into the mechanisms associated with HTP use is necessary; such studies should aim to expand the mechanistic pathways involved, drawing on established toxicological and pathophysiological frameworks.

## 5. Conclusions

Further research is needed to determine the short-term and long-term health effects associated with HTP products. There is a need for further longitudinal studies, mechanistic analyses, and independent studies independent of the tobacco sector. It is recommended that measures be taken to slow the growth in their adoption among younger people, currently the largest individual group of users, by organizing information campaigns and educational interventions about the health risks associated with HTP use.

There is also a need to introduce systems for evaluating novel tobacco products before they are introduced to the market, with the results being used to support policymakers in regulating HTP use to protect current nonsmokers.

## Figures and Tables

**Table 1:** Health effects of heated tobacco product use in human studies.

Author	Health Effects
**Respiratory system**	
	(+)
Sharman A, 2022, 2021 [38,46]	↓ lung function
Sakaguchi Ch, 2021 [39]	↓ plasma cotinine levels, ↓ NNAL (NTV compared to CC)
Polosa R, 2021 [45]	↓ annual COPD exacerbations, ↑ (improvement) CAT and 6MWD ↑ (improvement) respiratory symptoms, exercise tolerance, quality of life, and frequency of disease exacerbations
	(−)
Thomas M, 2024 [40]	abnormalities on chest radiograph, peripheral mild eosinophilia, pulmonary nodules, and ground-glass opacities on CT scan
Goebel I, 2023 [36]	↑ resistance and obstruction of small airways after consumption of substitute products and cigarettes
Majek P, 2023 [37]	↓ F_ENO_ fraction of nitric oxide in exhaled air
Zaitsu M, 2023 [41]	↑ frequency of allergies among offspring of current HTP smokers during pregnancy
Harada S, 2022 [42]	↓ FEV _1_
Kang BH, 2022 [43]	AEP on radiography, bilateral patches of pulmonary infiltrate, bilateral multifocal, patchy consolidations with multiple small nodular ground-glass opacities and thickened interlobar septa on computed tomography
Gülensoy SE, 2021 [44]	pleural atelectasia, fibro-atelectatic changes, pleural effusion, fibro-atelectatic changes in the lung lobe and pleural thickening in the left lung, nodular lymphoid aggregation, extensive anthracosis around the lung, fibrous material resembling a hyaline membrane in the alveoli, type 2 pneumocyte hyperplasia, interstitial organization, picture of subacute lung injury with exogenous lipid material
Sakaguchi Ch, 2021 [39]	↓ FEV1, %FEV1 levels among NTV users compared to NS group
**Cardiovascular system**	
	(+)
Sakaguchi Ch, 2021 [39]	↓TG, ↑ HDL-C, ↓sICAM-1, ↓WBC (NTV and CC)
Ikonomidis I, 2021 [58]	switching to HNBC improved CO (exhaled carbon monoxide), FMD, CFR, TAC, GLS, GWW, MDA, TxB2 ↓ effect on vascular and cardiac function of HNBC than tobacco cigarettes
	(−)
Antoniewicz Ł, 2025 [47]	↑ EVs of endothelial and platelet origin after short-term inhalation of HTP with nicotine
Znyk M, 2025 [48]	↑ PLT
Harada S, 2024 [49]	effects on the glutamate pathway, biomarkers involved in glutamate metabolism were associated with HTP use
Hu H, 2024 [50]	↑ risk of hypertension
Świątkowska B, 2024 [51]	↑ IL-8
Lyytinen G, 2024 [54]	↑ pulse wave velocity, ↑ blood pressure and heart rate, ↑ platelet clot formation, ↑ arterial stiffness
Belkin S, 2023 [52]	↑ white blood cell count, proinflammatory cytokines ↑ leukocytes, eosinophils, IL-6, IL-2, IL-8, ↑ inflammatory reaction followed by endothelial dysfunction ↑ arterial stiffness
Goebel I, 2023 [36]	↑ blood pressure and arterial stiffness
Koh DH, 2023 [53]	potential dose-response relationship with WBC counts in heated cigarette smokers
Majek P, 2023 [37]	↑ heart rate and blood pressure
Benthien J, 2022 [55]	↑ blood pressure and artery stiffness
Hu H, 2022 [56]	↓ HDL-C
Sharman A, 2021 [38]	↑ systolic blood pressure
Schirone L, 2022 [57]	TCC and HNBC similarly harmful for NO, H_2_O_2_, sCD40L, platelet aggregation, cotinine and FMD HNBC less harmful than TCC, for No Nox2-dp and P-selectin HNBCs harmful effects on oxidative stress and platelet activation
Sakaguchi Ch, 2021 [39]	↑ total NNAL, ↑ 2,3-d-TXB2
**Nervous system**	
	(−)
Lee BG, 2025 [59]	↑ anhedonia and depressed mood ↑ likelihood of moderate to severe depressive symptoms
**Oral cavity**	
	(+)
Gupta S, 2024 [60]	↑ Whiteness Index for Dentistry (WID)
Mišković I, 2024 [61]	↓ harmful effect on periodontal tissues (PD and CAL) compared to traditional cigarettes
Pouly S, 2021 [67]	↓ the risk of smoking-related diseases if beneficial changes are observed in the development of chronic generalized periodontitis after mechanical therapy
	(−)
Tadin A, 2024 [62]	↑ all parameters in the micronucleus test, except the number of cells with micronuclei, ↑ pyknotic cells, ↑ cytotoxic and genotoxic damage
Zięba S, 2024 [63]	HnB seem to have a similar mechanism of action on the immune system of unstimulated saliva, leading to the inhibition of the local inflammatory response in the oral cavity
Zięba S, 2024 [64]	↓ salivary lipid concentrations, ↑ MDA and 4-HNE concentration
Sever E, 2023 [65]	↓ SFR, ↑ halitosis, ↑ prevalence of intraoral findings ↑ dry mouth
Mori Y, 2022 [66]	↓ salivary secretion, ↓ salivary Lac, Lys secretion indices
**Reproductive system**	
	(−)
Incognito GG, 2024 [68]	↑ premature birth
Galanti F, 2023 [69]	↓ AMH, ↓ number of oocytes retrieved, ↑ number of empty oocytes with zona pellucida ↓ FR, ↓ reduced ovarian reserve and ovarian quality
Hosokawa Y, 2022 [70]	↑ incidence of SGA in women smoking HTP during pregnancy
**Metabolic system**	
	(−)
Jee Y, 2024 [71]	↑ metabolic risk
Hu H, 2023 [72]	↑ HbA1c and fasting glucose among exclusive HTP users, and dual users compared to never smokers ↑ likelihood of prediabetes and diabetes
Sharman A, 2022 [46]	↓ fasting blood glucose ↑ CAT results, waist circumference
**Biomarkers of exposure and biomarkers of effect**	
	(+)
Chu S, 2025 [73]	↓ BoE levels, no BoBE changes
Gale N, 2022 [74]	↓ BoE levels, several BoPH changed in a favorable direction (toward never-smoking levels) in participants who switched completely to HTP or quit smoking, BoPHs such as soluble intercellular adhesion molecule-1 changed in an unfavorable direction
Gale N, 2021 [75]	↓ most BoE levels in HTP users BoPH, including total 4-(methylnitrosamino)-1-(3-pyridyl)-1-butanol, 8-epi-prostaglandin F2α type III, fractional exhaled nitric oxide concentration, and white blood cell count, were directionally consistent with reduced health effects
**Molecular genetic effects**	
	(−)
Ohmomo H, 2022 [76]	↑ LRRN3 and GPR15 more hypomethylated in HTP users ↑ GPR15 expression HTP users show abnormal DNAm and transcriptome profiles
**Secondhand exposure**	
	(−)
Yoshioka T, 2023 [77]	↑ asthma attacks/asthma-like symptoms, ↑ persistent cough

Legend: (+) advantages, (−) disadvantages of using HTP; ↓ decrease, ↑ increase.

**Table 2:** Health effects of using heated tobacco products in vitro studies.

Human cells	Effect	Author
	(+)	
human lung fibroblast cells (MRC5)	ability to inhibit the growth of *S. pneumoniae*, *K. pneumoniae* minimal cytotoxicity to human lung fibroblasts reduced short-term toxicological profile	Furnari S, 2025 [78]
human microglial cells (HMC3)	HTP showed a lower degree of microglial toxicity, with reduced ROS production, lipid peroxidation, and mitochondrial dysfunction compared to traditional cigarettes	Distefano A, 2024 [79]
human periodontal ligament cells (hPDL)	HTP exposure led to a decrease in cell counts 48 h and 72 h after exposure HTP showed less harmful effects on hPDL compared to CS	Wiesmann-Imilowski N, 2024 [88]
human gingival fibroblasts and human keratinocytes	↑ cell viability (fibroblasts, keratinocytes), ↑ migration, ↑ number of cells in S and G2/M phase ↑ proliferation of oral cells	Pagano S, 2021 [90]
	(−)	
a human oral squamous cell carcinoma (OSCC) cell line, HSC-3	cytotoxic effect in OSCC cells exposure to CSE (HTP and traditional tobacco) from both sources led to an increase in intracellular Ca^2+^ concentration, induced p38 phosphorylation and cell apoptosis, and increased ROS levels	Kagemichi N, 2024 [80]
human bronchial epithelial BEAS-2B cells	3R4F and HTP emissions affected the profiles of exogenous compounds (one carcinogenic) and the profiles of endogenous metabolites from the following pathways: oxidative stress, energy metabolism, and lipid metabolism	Lenski M, 2024 [81]
human atrial cardiac stromal cells (CSCs)	cells cultured with HTP serum showed increased levels of profibrotic markers and decreased expression of connexin-43 TCC and HTP sera increased collagen release and decreased secretion of angiogenic protective factors from CSCs compared with NS serum circulating molecules in the serum of chronic exclusive HTP smokers induced fibrotic behavior in CSCs via activation of the mTOR pathway and decreased their beneficial paracrine effects on endothelial cells and cardiomyocytes	Picchio V, 2024 [86]
human lung adenocarcinoma (A549) cells with type II alveolar epithelial	HTP altered the CpG methylation pattern, affected gene expression, and affected mRNA expression and promoter methylation of CYP1A1, which is associated with carcinogenic risk	Sato A, 2023 [87]
gingival epithelial cells	Long-term HTP stimulation influenced epithelial differentiation and keratinization of gingival epithelial cells	Uehara O, 2023 [89]
human bronchial epithelial cells BEAS-2B	HTP was less cytotoxic than conventional cigarettes; HTP and cigarettes induced oxidative DNA damage and significantly increased DNA strand breaks and chromosomal aberrations.	Zarcone G, 2023 [82]
primary human oral keratinocytes (HOKs)	HTP increased the formation of γH2AX foci in HOK, and ATR ATR expression decreased in cells exposed to CSE with CC and HTP	Morishita Y, 2022 [83]
human models of bronchial and alveolar mucosa	↑ levels of total cellular reactive oxygen species, stress-responsive nuclear factor kappa-B, and DNA damage markers [8-hydroxy-2′-deoxyguanosine, phosphorylated histone H2AX, cleaved poly(ADP-ribose) polymerase] were detected in bronchial and/or alveolar models exposed to HTP smoke, ↑ IL1ꞵ and IL8 in the bronchial model, ↑ interferon-γ and IL4 in the alveolar model, ↓ IL13 in the alveolar model	Rahman M, 2022 [84]
endothelial cells	HTP treatment showed induction of HMOX1 and NQO1, ↓ VCAM1 expression in a dose-dependent manner, ↑ activation of antioxidant or pro-inflammatory patterns	Giebe S, 2021 [85]

Legend: (+) advantages, (−) disadvantages of using HTP; ↓ decrease, ↑ increase.

## Supplementary Materials

The following supporting information can be downloaded at: https://www.mdpi.com/article/10.3390/healthcare13162042/s1, Figure S1: Flow diagram outlining the studies included in the review; Table S1: Studies included in the review.

## Abbreviations

The following abbreviations are used in this manuscript:

FDA	Food and Drug Administration	%FEV1	% predicted FEV1
EU	European Union	CAT	COPD Assessment Test
WHO	World Health Organization	COPD	Chronic obstructive pulmonary disease
FCTC	Framework Convention on Tobacco Control	6MWD	6 min walking distance
HTP	Heated tobacco products	BoE	biomarkers of exposure
IQOS	I-Quit-Ordinary-Smoking	BoBEs	biomarkers of biological effect
THS	The Tobacco Heating System	DNAm	DNA methylation
PD	probing depth	LRRN3	DNAm biomarker genes
CAL	clinical attachment loss	GPR15	DNAm biomarker genes
HnB	heat-not-burn	WID	Whiteness Index for Dentistry
MDA	malondialdehyde	FR	fertilization rate
4-HNE	4-hydroxynonenal	HbA1c	hemoglobin A1c
SFR	Salivary flow rate	CT	Computed tomography
Lys	lysozyme secretion indicators	OSCC	oral squamous cell carcinoma
Lac	lactoferrin secretion indicators	CSE	cigarette-smoke-extract
CS	cigarette smokers	p38	protein
ICSI	Intracytoplasmic sperm injection	Ca^2+^	calcium ions
AMH	anti-Müllerian hormone	3R4F	reference cigarette smoke
SGA	small for gestational age	BEAS-2B	human bronchial epithelial cells
IL-8	interleukin (IL)-8	DSBs	double-strand breaks
IL-6	Interleukin (IL)-6	ATR-CHK1	ataxia telangiectasia, and Rad3-related (ATR) checkpoint kinase 1 (CHK1)
IL-2	interleukin (IL)-2	MDC1	Mediators of the DNA damage checkpoint 1
WBC	white blood cells	DNA	deoxyribonucleic acid
EVs	extracellular vesicles	NQO1	target genes
HNBC	Heat-Not-Burn cigarette	HMOX1	target genes
TCC	traditional combustion cigarette	VCAM1	Vascular cell adhesion molecule 1
Nox2-dp	Nox2-derived peptide	CSCs	human atrial cardiac stromal cells
ROS	reactive oxygen species	CpG	CpG (cytosine-guanine) dinucleotide in DNA
sCD40L	soluble CD40 ligand	RC	reference cigarette
H_2_O_2_	hydrogen peroxide	mRNA	messenger RNA
NO	nitric oxide	CYP1A1	cytochrome P450 family 1 subfamily Member 1
FMD	flow-mediated dilation	hPDL	human periodontal ligament cells
PLT	platelets	eCV	electronic cigarette vapor
HDL-C	high-density lipoprotein cholesterol	S and G2/M	S and G2/M cell cycle phases
NTV	novel tobacco vapor	p53, Bcl2, p21	genes
BoPH	biomarkers of potential harm	MRC5	human lung fibroblast cells
CC	conventional cigarette	HMC3	human microglial cells
NNAL	4-(methylnitrosamino)-1-(3-pyridyl)-1-butanol	HSC-3	human oral squamous cell carcinoma cell line
2,3-d-TXB2	2,3-dinor thromboxane B2	A549	human lung adenocarcinoma cells
NS	never-smokers	P450	cytochrome P450
CO	carbon monoxide	γH2AX	phosphorylated H2AX
CFR	coronary flow reserve	HOK	human oral keratinocytes
TAC	total arterial compliance	H2AX	histone H2A variant
GLS	global longitudinal strain	IL1ꞵ	Interleukin (IL)-1ꞵ
GWW	wasted myocardial work	IL4	Interleukin (IL)-4
TxB2	thromboxane B2	IL-13	Interleukin (IL)-13
AEP	acute eosinophilic pneumonia	PBMC	peripheral blood mononuclear cells
FEV 1	forced expiratory volume in 1 s	J-ECOH	the Japan Epidemiology Collaboration on Occupational Health
hs-CRP	high-sensitivity C-reactive protein	IL-16	Interleukin (IL)-16
NOS	the Newcastle-Ottawa Scale	HGF	cytokines
RCTs	Randomized controlled trials	Gro-α, MCP-1, SCF, MIG, IP-10	chemokines
NNK	biomarkers of 4-(methylnitrosamino)-1-(3-pyridyl)-1-butanone	G-CSF	granulocyte colony-stimulating factor
PR	the prevalence ratio	TRAIL	protein TNF-related apoptosis-inducing ligand, cytokine
IFNγ	interferon gamma	SFR	the salivary flow rate
GO	Gene ontology	OR	odds ratio
sICAM-1	soluble intercellular adhesion molecule-1	χ^2^	Chi-square test
HR	hazard ratio	NNN	*N*-nitrosonornicotine
sNox2-p	Serum levels of Nox2-derived peptide		
