# Supplementary material for: The Health Effects of Heated Tobacco Product Use—A Narrative Review
Source: Healthcare (Basel). 2025 Aug 18;13(16):2042. doi: 10.3390/healthcare13162042 (PMC12385413; doi:10.3390/healthcare13162042)
Supplement: Supplementary file 1 [file healthcare-13-02042-s001.zip › healthcare-3752040-supplementary.pdf]

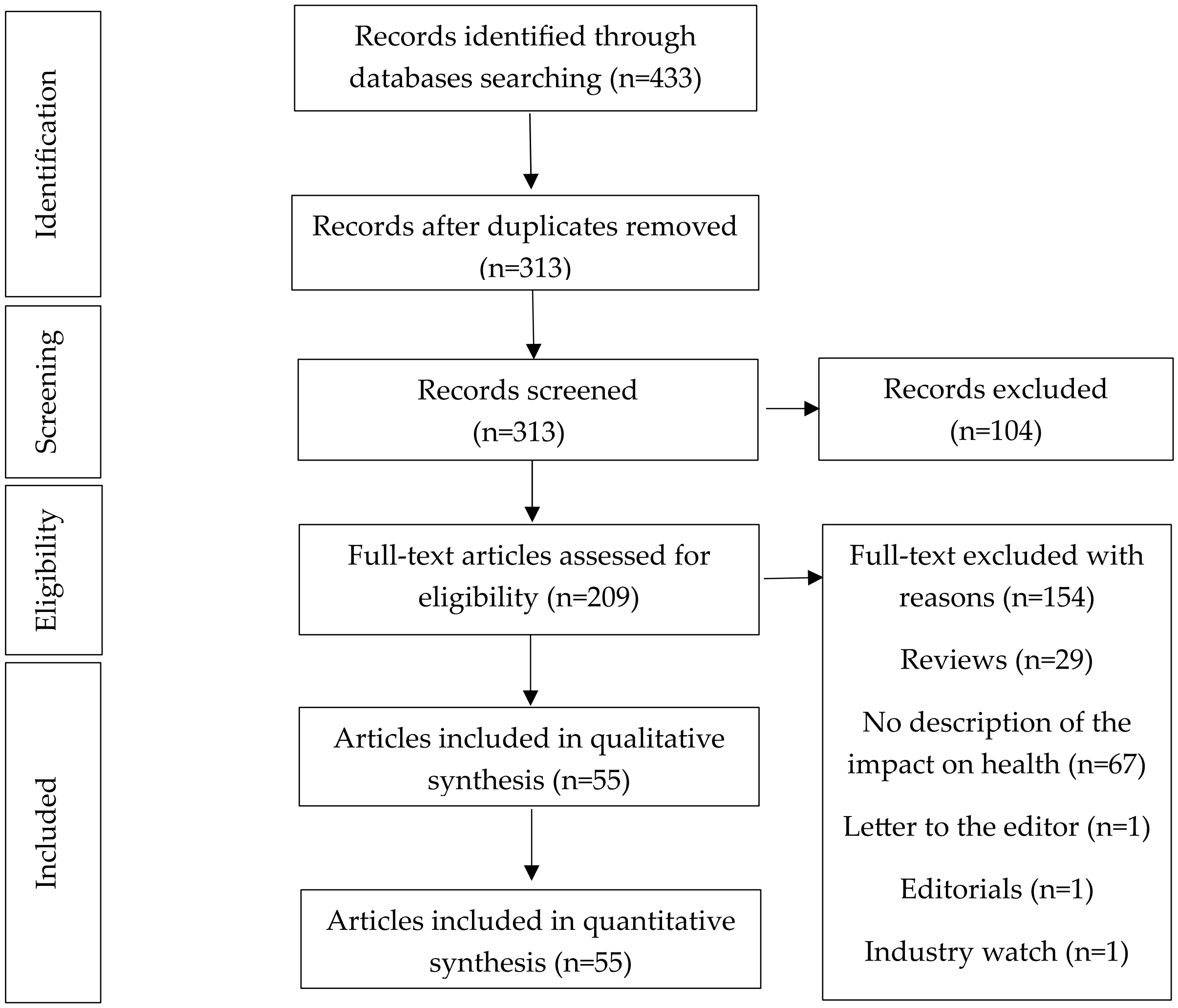

Figure S1. Flow diagram outlining the studies included in the review

Table S1. Studies included in the review

Authors, Year of Publication	Material/ Study design	Country	Funder	Main Objective
Antoniewicz Ł, 2025 [47]	human/ a randomized, crossover design	Sweden	independent	to investigate the acute effect of HTP inhalation on the level of extracellular vesicles (EV)
Chu S, 2025 [73]	human/ a randomized controlled trial	China	independent	effects of HTP on biomarkers and cigarette use patterns in adult smokers who do not want to quit
Furnari S, 2025 [78]	in vitro study	Italy	independent	to investigate the biological effects of HTP-generated aerosols, including their impact on the proliferation of two bacterial models, as the main factors of bacterial pneumonia
Lee BG, 2025 [59]	human/ a cross-sectional study	Republic of Korea	independent	establishing links between CC and HTP use and depressive symptoms
Znyk M, 2025 [48]	human/ a case-control study	Poland	independent	assessment of the effect of using heated tobacco on selected biochemical and morphological blood parameters
Distefano A, 2024 [79]	in vitro study	Italy	independent	assessment and comparison of the effects of cigarette smoke and HTP aerosol on microglia function, taking into account the toxicological profile, inflammatory response, and oxidative stress
Gupta S, 2024 [60]	human/ a cross-sectional study	Catania, Italy	independent	to show whether individuals who switched from smoking traditional cigarettes to heated tobacco products show better tooth colour indicators compared to regular tobacco smokers
Harada S, 2024 [49]	human/ a cohort study	Japan	independent	to investigate changes in metabolites associated with smoking among HTP users
Hu H, 2024 [50]	human/ a cohort study	Japan	independent	to investigate the relationship between cigarette and HTP use and the risk of hypertension

Incognito GG, 2024 [68]	human/ a retrospective, monocentric cohort study	Italy	independent	comparison of the effects of HTP or traditional cigarette use on maternal and neonatal outcomes
Jee Y, 2024 [71]	human/ a cohort study	South Korea	independent	investigate the effects of HTPs on the development of metabolic syndrome
Kagemichi N, 2024 [80]	in vitro study	Japan	independent	to investigate the cytotoxicity of CSE derived from HTP and traditional tobacco, taking into account the role of reactive oxygen species (ROS) and intracellular Ca^{2+}
Mišković I, 2024 [61]	human/ a cross-sectional study	Croatia	independent	assessment of the relationship between THS consumption and periodontal parameters
Lenski M, 2024 [81]	in vitro study	France	independent	comparison of the metabolome of human lung epithelial cells exposed to e-cigarette, HTP or 3R4F cigarette emissions to highlight potential early markers of toxicity
Picchio V, 2024 [86]	in vitro study	Italy	independent	to investigate the influence of circulating molecules in chronic HTP smokers on the fibrosis behavior of human cardiac stromal cells
Świątkowska B, 2024 [51]	human/ a case–control study	Poland	independent	to investigate the effect of HTP on biomarkers of inflammation in the blood
Tadin A, 2024 [62]	human/ a cross-sectional observational study	Croatia	independent	analysis of cytogenetic damage to the buccal mucosa in non-smokers, smokers of traditional combustible tobacco products, and non-combustible substitutes
Thomas M, 2024 [40]	case clinical	Katar	independent	a case of pulmonary complications associated with IQOS (a 40-year-old chronic smoker who switched to IQOS)
Wiesmann-Imilowski N, 2024 [88]	in vitro study	Germany	independent	to assess the proliferative capacity, metabolic activity, and potential damage of human periodontal ligament (hPDL) cells exposed to cigarette smoke, e-cigarette

				vapour, and heated HTP aerosol
Zięba S, 2024 [63]	human/ a case–control study	Poland	independent	to assess and compare the concentrations of a broad panel of cytokines, chemokines, and growth factors in unstimulated saliva samples from young smokers of traditional and electronic cigarettes, and tobacco heating systems, with the duration of addiction from one to three years.
Zięba S, 2024 [64]	human/case–control study	Poland	independent	to investigate the effects of smoking traditional cigarettes, e-cigarettes, and heat-not-burn cigarettes on the profiles of salivary lipids and lipid peroxidation products in unsimulated and stimulated saliva of healthy young adults with a smoking habit of up to 3 years
Belkin S, 2023 [52]	human/ a partly double-blinded, randomised, crossover trial	Germany	independent	to investigate the effects of acute consumption of combustible cigarettes, electronic cigarettes, and tobacco heaters on inflammation and arterial stiffness
Galanti F, 2023 [69]	human/ prospective observational longitudinal study	Italy	independent	to assess the impact of cigarette consumption and alternative devices (electronic and heat-not-burn products) on infertile women undergoing in vitro fertilization (IVF), in particular on the quality of oocytes collected from women undergoing intracytoplasmic sperm injection (ICSI) cycles
Goebel I, 2023 [36]	human/ a single-center, five-arm, crossover study	Germany	independent	acute effects of IQOS TM and glo TM consumption on small airway function and arterial stiffness
Hu H, 2023 [72]	human/ the Japan Epidemiology Collaboration on Occupational Health (J-ECOH) study	Japan	independent	analysis of the association between HTP use and prediabetes and diabetes

Koh DH, 2023 [53]	human/ a cross-sectional study	Korea	independent	to investigate the relationship between smoking heated cigarettes and the number of white blood cells
Majek P, 2023 [37]	human/ a intervention study	Poland	independent	to investigate the acute health effects on the respiratory and cardiovascular systems when using HTP
Lyytinen G, 2024 [54]	human/ a randomised crossover study	Sweden	independent	assessment of the effect of HTP use on vascular functions (arterial stiffness and platelet clot formation)
Sato A, 2023 [87]	in vitro study	Japan	dependent	evaluation of the effect of HTP on DNAm and gene transcription in human lung epithelial cells in vitro
Sever E, 2023 [65]	human/ a cross-sectional study	Croatia	independent	analysis and comparison of the effects of traditional cigarettes and THS on oral mucosa, salivary flow rate (SFR), halitosis, and Candida spp. burden
Uehara O, 2023 [89]	in vitro study	Japan	independent	the effect of Ploom TECH + extract on gingival epithelial cell
Yoshioka T, 2023 [77]	human/ a cross-sectional study	Japan	independent	to investigate the association between passive exposure to HTP aerosol and respiratory symptoms (asthma attacks/asthma-like symptoms and persistent cough)
Zaitsu M, 2023 [41]	human/ a cross-sectional study	Japan	independent	to determine whether maternal HTP smoking is associated with allergy in her offspring and to assess the potential dose–response relationship
Zarcone G, 2023 [82]	in vitro study	France	independent	comparison of the effects of e-cigarette, HTP, and conventional cigarette emissions on the generation of oxidative stress and genetic and epigenetic changes in human bronchial epithelial cells BEAS-2B
Benthien J, 2022 [55]	human/ a single-center, randomized, four-arm	Germany	independent	to examine the acute effects of the JUUL device on peripheral blood pressure, arterial

	study with a crossover design			stiffness, endothelial vasodilator function, and inflammatory response
Gale N, 2022, 2021 [74,75]	human/ a randomised, controlled, parallel group, open-label, ambulatory clinical study	Great Britain	independent	to investigate whether biomarkers of exposure (BoE) and potential harm (BoPH) change when smokers either continue smoking or switch from cigarette smoking to exclusive use of HTP in an outpatient setting over 1 year
Harada S, 2022 [42]	human/ a longitudinal cohort study	Japan	independent	to investigate the effect of HTP use on the decrease in forced expiratory volume in one second (FEV1)
Hu H, 2022 [56]	human/ J-ECOH study	Japan	independent	analysis of the relationship between HTP use and HDL-C concentration
Hosokawa Y, 2022 [70]	human/ a cross-sectional study	Japan	independent	analysis of the association between HTP use during pregnancy and the prevalence of SGA (gestational age)
Kang BH, 2022 [43]	case clinical	Kaunas, Lithuania	independent	a case of acute eosinophilic pneumonia (a 22-year-old woman smoked HTP for 2 weeks, and just before symptoms appeared, she increased the dose from 6 per day to 15 per day)
Mori Y, 2022 [66]	human	Japan	independent	comparison of lactoferrin (Lac) and lysozyme (Lys) secretion indices in saliva of HnB smokers and non-smokers
Morishita Y, 2022 [83]	in vitro study	Japan	independent	comparison of cytotoxic, genotoxic, and toxicogenomic effects of HTP and CC in carcinogenesis through multi-step gene mutations in oral mucosa cells
Ohmomo H, 2022 [76]	human/ a cohort study	Japan	independent	DNAm and transcriptome analysis, showing the effect of HTP exposure on smoking-related DNAm biomarkers and gene expression changes in peripheral blood mononuclear cells (PBMC)

Rahman M, 2022 [84]	in vitro study	Sweden	independent	to investigate the toxicological response to HTP smoke exposure in the bronchial and alveolar regions of the lungs
Sharman A, 2022, 2021 [46,38]	human/ a cohort study	Kazakhstan	independent	analysis of the long-term effects of switching to HTP in long-term smokers compared to continuous use of traditional cigarettes
Schirone L, 2022 [57]	human/ a prospective observational cross-sectional study	Italy	independent	assessment of the effect of chronic TCC use compared to HNBC, taking into account oxidative parameters, platelet aggregation, and vascular parameters
Giebe S, 2021 [85]	in vitro study	Germany	independent	analysis of endothelial function parameters after exposure to aqueous smoke extracts (AqE) from HTP, e-cig, conventional cigarette (3R4F), and pure nicotine
Gülensoy SE, 2021 [44]	case clinical	Türkiye	independent	a case of chronic obstructive lung disease, coronary artery disease, and previous pulmonary embolism (a 56-year-old male patient had been using IQOS for 2.5 years).
Ikonomidis I, 2021 [58]	human/ an acute, randomised, crossover trial	Greece	independent	to assess the effect of HNBC on endothelial function, arterial stiffness, myocardial deformation, oxidative stress, and platelet activation in acute conditions and 1 month after switching to HNBC smoking, in comparison with traditional tobacco cigarettes
Pagano S, 2021 [90]	in vitro study	Italy	independent	to investigate the biological effects of IQOS smoking on human gingival fibroblasts and human keratinocytes by analyzing cell viability, morphology, migration, apoptosis, and cell cycle
Polosa R, 2021 [45]	human/ a retrospective-prospective observational study	Italy	independent	assessment of health parameters in a cohort of COPD patients who used HTP daily

Pouly S, 2021 [67]	human/ a randomized controlled two-arm parallel-group multicenter study	Japan	dependent	to demonstrate beneficial changes in periodontal endpoints in response to mechanical periodontal treatment in patients with generalized chronic periodontitis who switched completely to THS compared to continued smoking
Sakaguchi Ch, 2021 [39]	human/ an observational, cross-sectional, three-group, multi-center study	Japan	independent	to investigate the effect of biomarkers of potential harmfulness (BoPH) of one of the HNBP, under the conditions of actual use
